# Mechanisms Generating Cancer Genome Complexity: Back to the Future

**DOI:** 10.3390/cancers12123783

**Published:** 2020-12-15

**Authors:** Franck Toledo

**Affiliations:** Genetics of Tumor Suppression, Institut Curie, PSL Research University, Sorbonne University, CNRS UMR3244 Dynamics of Genetic Information, 26 rue d’Ulm, CEDEX 05, 75248 Paris, France; franck.toledo@curie.fr; Tel.: +33-(0)1-56-24-66-71

**Keywords:** breakage-fusion-bridge cycles, micronuclei, chromothripsis, cancer genome evolution, gene amplification, telomeres, p53

## Abstract

**Simple Summary:**

In the 1990s, fluorescent in situ hybridization approaches made it possible to analyze the early stages of gene amplification in mammalian cells. These studies established breakage-fusion-bridge cycles as a major mechanism of intrachromosomal gene amplification. They also revealed that the amplified DNA perturbed nuclear architecture and led to micronucleation, which suggested a mechanism for the shortening of amplified units. The “interphase breakage model” postulated that the tremendous genomic instability occurring at early stages of gene amplification resulted from the interweaving of an amplification mechanism (breakage-fusion-bridge cycles) and of a deletion mechanism (micronucleation and stitching of DNA fragments retained in the nucleus). This model is strikingly consistent with recent data and conclusions obtained from live-cell imaging and single cell genome sequencing. The comparison of both sets of data suggests new questions to explore.

**Abstract:**

Understanding the mechanisms underlying cancer genome evolution has been a major goal for decades. A recent study combining live cell imaging and single-cell genome sequencing suggested that interwoven chromosome breakage-fusion-bridge cycles, micronucleation events and chromothripsis episodes drive cancer genome evolution. Here, I discuss the “interphase breakage model,” suggested from prior fluorescent in situ hybridization data that led to a similar conclusion. In this model, the rapid genome evolution observed at early stages of gene amplification was proposed to result from the interweaving of an amplification mechanism (breakage-fusion-bridge cycles) and of a deletion mechanism (micronucleation and stitching of DNA fragments retained in the nucleus).

## 1. Introduction

In a recent issue of Science, Umbreit et al. used live-cell imaging with single cell whole genome sequencing (Look-Seq) to analyze the cascade of genome rearrangements following the formation of a chromosome bridge in human cells [1]. Their results led to propose that this bridge leads to a first breakage-fusion-bridge (BFB) cycle—a mutational process initially proposed by Barbara McClintock [2]—but that additional BFB cycles are interwoven with episodes of micronucleation and chromothripsis—a catastrophic event resulting from “the shattering of a chromosome or chromosomal region into tens to hundreds of pieces, some of which are stitched together in a mosaic patchwork of genomic fragments” [3]. Umbreit et al. concluded that interwoven BFB cycles, micronucleation and chromothripsis episodes may underly the ongoing evolution of cancer genomes. This conclusion is strikingly consistent with the “interphase breakage model” proposed almost 30 years ago [4]. Below is a recollection of the fluorescent in situ hybridization (FISH) data that led to propose the interphase breakage model, and a discussion of this model in the light of more recent studies.

## 2. Early Stages of Gene Amplification are Characterized by Extreme Genomic Instability

**Figure 1:**
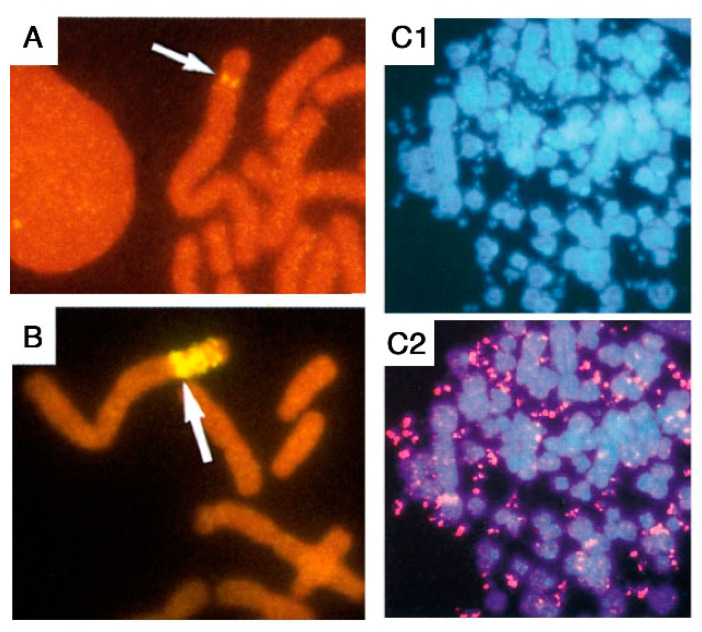
Gene amplification in mammalian cells can be intra- or extra-chromosomal. Fluorescent in situ hybridization (FISH) with a probe specific to the *AMPD2* gene was performed in cells either unamplified (**A**) or exhibiting intra- (**B**) or extra-chromosomal (**C**) *AMPD2* gene amplification. Partial metaphases are shown. In (**A**,**B**), a biotinylated probe was hybridized to chromosomes stained in red with propidium iodide, and the *AMPD2* genes (arrows) were detected with avidin and anti-avidin antibodies coupled with FITC (yellow fluorescence). In (**C**), a digoxigenin-labelled probe was hybridized to chromosomes and DMs stained in blue with DAPI (**C1**), and the *AMPD2* genes were detected (**C2**) with antibodies against digoxigenin coupled with TRITC (red fluorescence). Adapted from refs. [6,7].

Genome rearrangements are frequently observed in tumor cells, encompassing net gains or losses of whole chromosomes (aneuploidy) or parts of chromosomes (deletions, non-reciprocal translocations, gene amplification) that may include complex events such as chromothripsis. Gene amplification, defined as a copy number increase of a restricted region of a chromosome, may contribute to tumor progression by increasing the copy number, and thereby the expression, of oncogenes [5]. The amplified DNA can be organized as intrachromosomal extra-copies that may form a cytologically observable homogeneously staining region (HSR), or as extra-chromosomal copies called double minutes (DMs) (Figure 1).

Efforts to understand the mechanisms underlying oncogene amplification were initially hampered by the high level of genome complexity in cells recovered from advanced tumors. However, in the 1990s, several groups gained insights into these mechanisms by using model systems of cultured cells selected for resistance to a cytotoxic drug. Essential for these advances were the development of the FISH technique that made it possible to analyze the amplified DNA at the level of a single cell, and the design of experimental protocols allowing the recovery of mutant cells 10–35 generations after initiation of the amplification process. The early mutants analyzed were cells amplified for the *DHFR* gene, which confers resistance to methotrexate [8,9,10,11]; the *CAD* gene, which confers resistance to phosphonacetyl-aspartate [12,13,14]; the *AMPD2* gene, which confers resistance to coformycin [4,6,7,11,15] and the *MDR1* gene, which confers resistance to drugs including vinblastin, adriamycin and actinomycin D [7,11]. FISH with a probe for the amplification “driver” gene (conferring drug resistance) first revealed that early amplified units, within HSRs or DMs, were often tens of Megabases (Mb) in length, suggesting that their amplification resulted from an uneven segregation of driver sequences during successive cell cycles, rather than local over-replication [6,8,9,12]. Furthermore, these studies revealed a transient, but extreme genomic instability at early stages of the amplification process. For example, when clones were analyzed 20 cell divisions after initiation of *AMPD2* gene amplification, the distribution of intrachromosomal *AMPD2* extra-copies varied from cell to cell within a single clone, and one third of the cells exhibited karyotypic abnormalities such as dicentric and ring chromosomes. By contrast, clones analyzed 10 cell divisions later were more homogeneous and exhibited fewer karyotypic anomalies. The comparison of these two sets of clones suggested that chromosomes with 2–4 *AMPD2* copies separated by ca. 45 Mb corresponded to the earliest amplified structures, which were progressively overtaken by structures with more units of reduced and irregular sizes [6]. However, the precise mechanisms underlying gene amplification or the rapid shortening of amplified units remained unknown.

## 3. A Major Role for Breakage-Fusion-Bridge Cycles in Gene Amplification in Mammalian Cells

**Figure 2:**
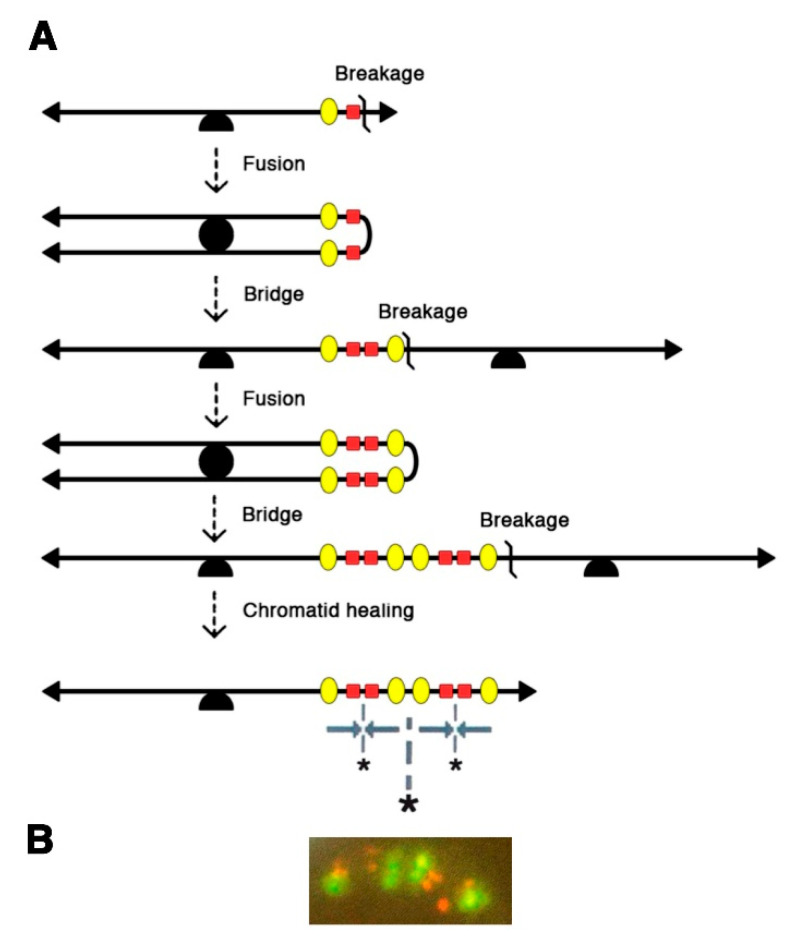
Chromatid Breakage-Fusion-Bridge cycles account for the symmetrical structures observed at early stages of gene amplification in mammalian cells. (**A**) At the earliest stages of gene amplification, breakage-fusion-bridge (BFB) cycles of sister chromatids lead to megabase-long palindromes with one or two orders of symmetry, containing equal copy numbers of the amplification driver gene (conferring drug resistance; red squares) and of the passenger marker (yellow ovals, centromeric to the driver in an unamplified chromosome). Grey arrows: orientation of amplified units; asterisks: symmetry axes; arrowheads: telomeres; half-circle: centromere. (**B**) Example of a palindromic structure observed on an *AMPD2*-amplified metaphase chromosome. This structure was revealed by two-color FISH with a digoxigenin-labelled probe for the amplification driver *AMPD2* (in red) and a biotinylated probe for P3C4, a passively co-amplified marker (in yellow). Adapted from Figures 2 and 4 of Ref. [4].

A better understanding of the mechanisms involved came from two-color FISH analyses, with one probe for *AMPD2*, the amplification “driver” gene, and one probe for a passively co-amplified (“passenger”) marker [4]. These experiments showed that the early intrachromosomal amplified units were organized as Mb-long inverted repeats with one or several orders of symmetry, which were perfectly explained by the operation of breakage-fusion-bridge cycles between sister chromatids (Figure 2). According to this model, which B. McClintock initially described as the chromatid type of BFB cycles, the sister-chromatids produced after replication of a broken chromatid would fuse at the location of the break, hence generating a dicentric chromatid. At anaphase, the centromeres of the dicentric chromatid would move to opposite poles of the mitotic spindle, creating a bridge which is later broken, and this may initiate another cycle of fusion, bridge and breakage, until some process, unknown at that time, heals the broken chromatid [2]. B. McClintock obtained evidence of this mechanism in maize cells, by observing its “bridge” and “breakage” intermediates [16], and later reported the “fusion” intermediate [17]. Likewise, at early stages of mammalian *AMPD2* gene amplification, the palindromes with multiple levels of symmetry were expected products of chromatid BFB cycles [4] and the “bridge” and “fusion” intermediates, identified later [15], provided further evidence for this mechanism. The expected products and/or intermediates of chromatid BFB cycles were also observed in cells amplified for the *DHFR* or *MDR1* genes [10,11]. Furthermore, evidence for a related mechanism, the chromosome type of BFB cycles involving dicentric and ring chromosomes [2], was found in cells amplified for the *CAD* [13] or *AMPD2* [15] genes. In *AMPD2*-amplified cells, chromosome BFB cycles were shown to result from secondary mutational events occurring in cells already undergoing chromatid BFB cycles [15]. Consistent with this, in another model system, BFB cycles affecting one chromosome were found to subsequently cause the instability of multiple chromosomes through translocations [18]. Together, these studies revealed BFB cycles as a major mechanism of mammalian gene amplification and chromosomal instability.

These results implied that a single double-strand break could initiate the gene amplification process [4,10,15]. Alternatively, dysfunctional telomeres might also initiate intrachromosomal amplification [13,19]. In support of the hypothesis that DNA breaks may initiate gene amplification, B. McClintock initially observed BFB cycles resulting from double-strand breaks caused by transposition events or by γ-irradiation [20]. Furthermore, the drugs methotrexate, phosphonacetyl-aspartate or coformycin, used to select cells amplified for the *DHFR*, *CAD* or *AMPD2* genes, are known DNA damaging agents. Interestingly, despite the extreme genomic instability at early stages of *AMPD2* gene amplification, amplified units of similar sizes were observed in independent *AMPD2*-amplified clones [6], which indicated that the earliest cyclic breaks might occur at specific “fragile” sites, rather than at random locations [21], a possibility also suggested by the structure of *CAD*-amplified chromosomes [14]. In that regard, the ability to use several drugs to select for *MDR1* gene amplification provided crucial information [11]. Clones with *MDR1* gene amplification were selected for with adriamycin or actinomycin D, which are both DNA damaging drugs, or with vinblastine, which only acts as a spindle poison at lower doses. Surprisingly, *MDR1*-amplified clones were recovered infrequently upon selection with adriamycin or vinblastin and exhibited either intrachromosomal or extrachromosomal amplified copies, whereas clones with *MDR1* gene amplification were much more frequent upon selection with actinomycin D and always resulted from chromatid BFB cycles [11]. The difference between the two DNA damaging drugs could be explained by the fact that adriamycin induced breaks at random locations, whereas actinomycin D caused breaks at specific chromosomal loci defined as “common fragile sites,” which are regions where gaps and breaks are detected at high frequency in metaphase chromosomes when cells are grown under conditions causing DNA replication stress [11]. Further analyses showed that methotrexate and coformycin were also inducing breaks at common fragile sites, and suggested that the ability of a drug to induce the amplification of a given gene relied on its ability to activate a fragile site telomeric to the gene. Furthermore, fragile sites flanking *AMPD2* were found to determine the content of the earliest amplified units, accounting for units of similar sizes in independent cellular clones [11].

These conclusions relied on analyzing model systems of gene amplification, but they rapidly appeared relevant to understand the instability of cancer genomes. In the late 1980s, Hartwell and Weinert discovered the existence of cell cycle checkpoints in yeasts, and proposed that similar checkpoints might be important in mammals, particularly to ensure proper embryonic development [22]. A few years later, the observation that gene amplification in mammalian cells relies on cyclic DNA breaks [4], and that p53 controls the G1-S checkpoint [23] and prevents gene amplification [24,25], led Hartwell to propose that “defects in a cell cycle checkpoint may be responsible for the genomic instability of cancer cells” [26]. Furthermore, megabase-long inverted repeats were soon found in tumor cells amplified for various oncogenes (e.g., *CCND2*, *RIN1*, *MET*, *PIP*, *CCND1* or *ERBB2* [27,28,29,30,31,32]), while bridge intermediates were reported in tumors amplified for *MDM2* or the 11q13 chromosomal region [33,34], and chromatid fusions were observed in tumor cells amplified for *MYC* [35]. Moreover, telomere attrition caused BFB cycles and cancers in p53 mutant mice deficient for telomerase [36], and mice deficient for both p53 and a nonhomologous end joining DNA repair protein developed lymphomas with BFB cycles leading to *MYC* gene amplification [37]. Furthermore, evidence that the boundaries of amplified units in cancer cells might rely on breaks occurring at common fragile sites came first from analyzing reports in the Genome Data Base [11], then from experimental data [28,29,30,31,38,39,40,41]. Finally, hypoxia, a frequent property of the solid tumor microenvironment, was found to cause breaks at chromosome fragile sites and to promote the rearrangement of the amplified DNA [7].

Interestingly, in both the *AMPD2* and *MDR1* model systems, extrachromosomal amplification of the selected gene on DMs was observed in a few drug-resistant clones, and best explained by the looping-out of a circular molecule containing the selected gene, followed by an unequal segregation of circular molecules at subsequent mitoses [11,15]. The initiation of this amplification process is independent from BFB cycles, but a later reintegration of DMs into chromosomes may trigger secondary BFB cycles [7]. In sum, although the genome instability of cancer cells often mask the initial mechanisms responsible for oncogene amplification, evidence of BFB cycles could still be observed in some tumors, either because some early structures are selected for during the clonal expansion of tumors cells [30], or because BFB cycles might be a late rather than an initial mechanism of gene amplification in some cases [7]. In recent years, the analysis of cancer genomes shifted from cytogenetics to high-throughput genome sequencing, and algorithmic approaches to detect BFB cycles in tumor genomes were implemented [42,43,44]. With these approaches, BFB cycles appeared to be enriched in esophageal, lung and head and neck squamous cell cancers, and to be mostly implicated in the amplification of the *CCND1*, *ERBB2* and *CCNE1* oncogenes [45]. Importantly, prior FISH studies had reported amplification of these three genes in cancer cells, and provided evidence that BFB cycles with breaks at fragile sites or complex fragile regions might underly the amplification of *CCND1* [11,28,31] and *ERBB2* [32,46].

## 4. Further Rearrangements of the Amplified DNA: The Interphase Breakage Model

**Figure 3:**
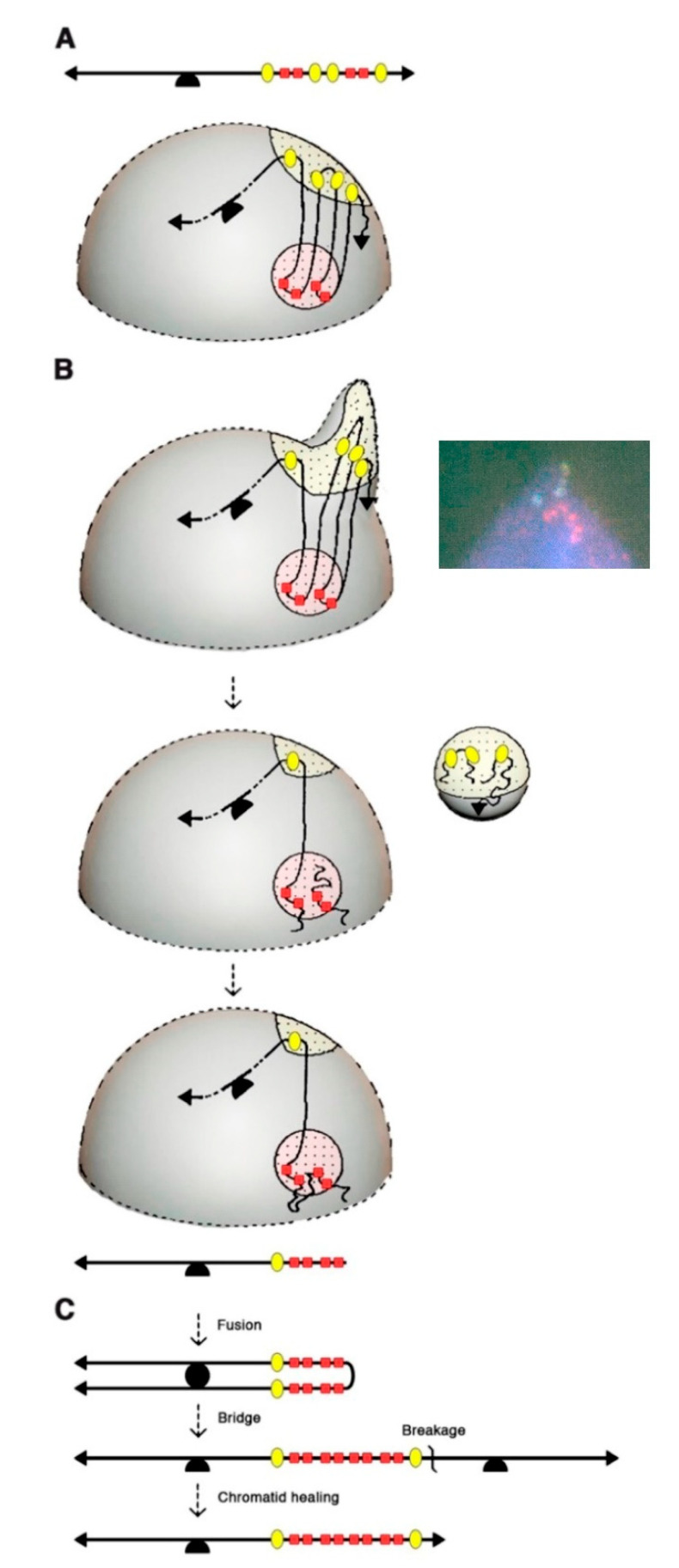
The interphase breakage model, integrating micronucleation, multiple DNA breaks and BFB cycles, can explain the rapid evolution of amplified chromosomes. (**A**) Co-amplified markers alternating in metaphase chromosomes (top) may segregate in interphase nuclei (bottom) into distinct nuclear domains (dotted areas). Red squares: amplification driver gene; yellow ovals: passenger marker; arrowheads: telomeres; half-circle: centromere. (**B**) The co-amplification of sequences belonging to distinct nuclear domains may perturb nuclear architecture, and lead to the extrusion of passenger sequences in a micronucleus. Micronucleus extrusion is associated with multiple DNA breaks, and the DNA fragments retained in the nucleus are stitched together to generate an amplified chromatid with an excess of driver sequences. (**C**) The amplified chromatid lacks a telomere and may undergo further BFB cycle(s). In (**B**) a typical nuclear malformation from an AMPD2-amplified cell analyzed by FISH is shown. Extra-copies of *AMPD2* (in red) and P3C4 (in yellow) segregate into distinct domains, and the nuclear bulge only contains P3C4 copies. Adapted from Figures 3 and 5 of Ref. [4].

BFB cycles explained the intrachromosomal amplification of the *AMPD2* gene, in particular the presence of inverted repeats, several tens of Mb in length, with several orders of symmetry. However, the observation of much shorter units very early in the amplification process suggested the possible implication of another mechanism [4]. Evidence for such a mechanism came from four distinct observations: (1) driver and passenger amplified copies alternated in Mb-long inverted repeats on metaphase chromosomes, but unexpectedly, the amplified copies of each marker often segregated into distinct nuclear domains in interphase nuclei (Figure 3A); (2) unlike cells from the parental cell line, 35% of the amplified cells exhibited nuclear malformations, ranging from nuclei with small bulges, to nuclei with large blebs or releasing micronuclei; (3) 80% of the nuclear malformations contained amplified DNA, and in most cases extra-copies of only one marker; (4) the chromosomes with larger amplified units exhibited equal numbers of driver and passenger sequences, but chromosomes with shorter units often had many copies of the driver flanked by only two copies of the passenger. Together, these observations indicated that the amplified DNA perturbed nuclear architecture, and further suggested that multiple DNA breaks occurring in a single interphase could lead to the extrusion of amplified passenger sequences in a micronucleus, and that driver-containing DNA fragments retained in the nucleus could then be stitched together to generate an amplified chromatid with shorter amplified units (Figure 3B). This chromatid might have a broken end and undergo further BFB cycle(s), leading to a chromatid with many copies of the driver flanked by two copies of the passenger (Figure 3C).

According to this “interphase breakage model,” the tremendous genomic instability occurring at early stages of mammalian gene amplification would result from the interweaving of an amplification mechanism (BFB cycles) and of a deletion mechanism (micronucleation and stitching of DNA fragments retained in the nucleus) [4]. Furthermore, the interphase breakage model was also proposed to contribute to the frequent observation of dicentric chromosomes at early stages of gene amplification, in diploid cells if sequences from two distinct chromosomes were simultaneously extruded in a micronucleus or in polyploid cells assuming that the micronucleation process favors endo-mitotic reduplication by affecting the integrity of the nuclear membrane [15].

Again, the interphase breakage model relied on the analysis of cells resistant to a cytotoxic drug, but evidence of its potential relevance to cancer cells was soon reported. Cells from well-differentiated liposarcomas, which often exhibit abnormal chromosomes (ring or giant rod chromosomes) with amplification of the 12q13-14 region, displayed blebs and micronuclei carrying DNA from the abnormal chromosomes [47,48]. Furthermore, evidence of a relationship between bridge-breakage of a chromosome and interphase bleb or micronuclei carrying sequences of the same chromosome was reported in malignant fibrous histiocytoma cells [49]. Interestingly, in neuroblastoma cells carrying *MYCN* extrachromosomal amplified copies, nuclear blebs and micronuclei containing *MYCN* copies were reported [50], raising the possibility that the interphase breakage model might not be restricted to cells with HSRs, but could also apply to cells with DMs.

The interphase breakage model, which postulates micronucleation-induced multiple DNA breaks followed by the stitching together of DNA fragments that remain in the nucleus, can be considered as a foreshadow to chromothripsis, identified 20 years later through whole genome sequencing [3]. Interestingly, the sequestration of an anaphase lagging chromosome into a micronucleus was found to cause chromothripsis of the isolated chromosome [51,52]. However, the latter mechanism would best explain the shattering of an entire chromosome, rather than chromothripsis events limited to a chromosomal region (e.g., an HSR). Furthermore, the interphase breakage model postulated a link between BFB cycles and multiple simultaneous DNA breaks in interphase, and accordingly, several cancer genomic analyses later suggested a link between BFB cycles and chromothripsis [53,54,55]. Also consistent with this, dicentric chromosomes formed during telomere crisis correlated with chromothripsis events in post-telomere crisis cells [56]. In their recent study, Umbreit et al. found chromosome bridges to induce micronucleation, not immediately after breakage of the chromosome bridge, but rather in the second cell generation, and this correlated with a massive increase in chromothripsis events [1]. This appears again consistent with FISH data, because in the *AMPD2* system, amplified chromosomes with 2–4 copies of the *AMPD2* (driver) gene exhibited the same copy number of passenger sequences, and thus, could be explained by one or two BFB cycles (Figure 1), whereas chromosomes with 5–15 *AMPD2* copies exhibited fewer copies of the passenger marker (Figure 2), suggesting that micronucleation and multiple interphase breaks occurred subsequently to the first or second BFB.

## 5. Conclusions and Future Perspectives

FISH studies and the more recent Look-Seq analyses have provided remarkably consistent data, leading to conclude that BFB cycles, micronucleation and multiple interphase DNA breaks are interwoven to drive the evolution of cancer genomes. However, while both sets of studies suggested that an amplified chromosome may give rise to a micronucleus, the proposed mechanisms for micronucleation appear to differ: in the interphase breakage model, micronucleation would result from a nuclear blebbing of the amplified DNA, whereas Umbreit et al. proposed that a micronucleus might be formed around a lagging broken chromosome. Further analyses will thus be required to precisely define the mechanisms underlying micronucleus formation. In addition, each approach generated unique information. For example, it was first assumed that spindle forces at mitosis caused the breakage of chromosome bridges in mammalian cells [57]—as initially reported in maize cells [2]—but live cell imaging revealed that chromosome bridges may persist for several hours in interphase [1,56] and suggest a critical role for cytoplasmic actomyosin contractile forces in chromosome bridge breakage [1]. Furthermore, Look-Seq approaches revealed that chromosome bridges and micronuclei may undergo unexpected bursts of DNA replication that might contribute to chromothripsis [1], and whole genome sequencing provided evidence for a prominent role of the cytoplasmic exonuclease TREX1 in chromothriptic DNA fragmentation [58]. On the other hand, FISH studies revealed the segregation of co-amplified markers into distinct nuclear domains, an observation that could not be made with Look-Seq approaches and that was crucial to suggest the interphase breakage model. These co-amplified markers might segregate in interphase because they belong to distinct topologically associated domains [59], or to distinct nuclear subcompartments [60], which could also explain why their co-amplification can perturb nuclear architecture. This suggests that a better understanding of nuclear organization might provide additional clues about the mechanisms underlying complex cancer genome rearrangements. In this regard, the distribution of chromothripsis events [55] and chromatin domains [61] was recently reported for the same 2658 tumors, and integrating both analyses might be useful. Finally, the computational classification of complex structural variants in cancer genomes is still evolving. Complex structural variant patterns were proposed to result from BFB cycles [43], chromothripsis [3,62], chromoplexy [63] and templated insertion chains [64,65], but considering that BFB cycles and chromothripsis events may be interwoven should add further complexity. Interestingly, the Pan-Cancer Analysis of Whole Genomes Consortium recently classified chromothripsis events from 587 tumors into five categories, and two of them (“liposarcoma-like chromothripsis” and “amplified chromothripsis”) show substantial overlap with BFB cycles according to a computational analysis by Hadi et al. [45,66]. Moreover, three new complex structural variant patterns were recently reported from analyzing junction copy number (JCN) in cancer genomes: pyrgo, rigma and tyfonas [45]. Pyrgo are “towers” of low-JCN duplications associated with early-replicating regions and superenhancers, rigma comprise “chasms” of low-JCN deletions enriched in late-replicating fragile sites, and tyfonas are “typhoons” of high-JCN and fold-back inversions. Among these three new patterns, tyfonas share many features expected from the operation of BFB cycles (high JCN in *cis*, with a high proportion of fold-back inversion junctions), but with additional complexity. Tyfonas were found in 80% of dedifferentiated liposarcomas and are thought to represent the genomic footprint of supernumerary ring chromosomes in this tumor type [45]. Because FISH studies provided evidence of BFB cycles, nuclear blebbing and micronucleation in well-differentiated liposarcomas [47,48] (carrying genetic aberrations also found in dedifferentiated liposarcomas [67]), it will be important to determine whether or not tyfonas may result from events described in the interphase breakage model.
